# High bioreactor production and emulsifying activity of an unusual exopolymer by *Chromohalobacter canadensis* 28

**DOI:** 10.1002/elsc.202000012

**Published:** 2020-06-02

**Authors:** Nadja Radchenkova, Ivanka Boyadzhieva, Merve Erginer Hasköylü, Nikolina Atanasova, Songül Yaşar Yıldız, Margarita J. Kuncheva, Ivan Panchev, Hristo Kisov, Spasen Vassilev, Ebru Toksoy Oner, Margarita S. Kambourova

**Affiliations:** ^1^ The Stephan Angeloff Institute of Microbiology Bulgarian Academy of Sciences Sofia Bulgaria; ^2^ IBSB, Department of Bioengineering, Faculty of Engineering Marmara University Istanbul Turkey; ^3^ Department of Bioengineering, Faculty of Engineering and Natural Sciences Istanbul Medeniyet University Istanbul Turkey; ^4^ Departments of Organic Chemistry and Physics University of Food Technologies Plovdiv Bulgaria; ^5^ Institute of Optical Materials and Technologies Bulgarian Academy of Sciences Sofia Bulgaria

**Keywords:** agitation and aeration, biocompatibility, emulsifying properties, exopolymer, halophilic bacterium

## Abstract

Unusual composition of an exopolymer (EP) from an obligate halophilic bacterium *Chromohalobacter canadensis* 28 has triggered an interest in development of an effective bioreactor process for its production. Its synthesis was investigated in 2‐L bioreactor at agitation speeds at interval 600‐1000 rpm, at a constant air flow rate of 0.5 vvm; aeration rates of 0.5, 1.0, and 1.5 vvm were tested at constant agitation rate of 900 rpm. EP production was affected by both, agitation and aeration. As a result twofold increase of EP yield was observed and additionally increased up to 3.08 mg/mL in a presence of surfactants. For effective scale‐up of bioreactors mass transfer parameters were estimated and lowest values of *K_L_a* obtained for the highest productivity fermentation was established. Emulsification activity of EP exceeded that of trade hydrocolloids xanthan, guar gum, and cellulose. A good synergism between EP and commercial cellulose proved its potential exploration as an enhancer of emulsifying properties of trade emulsions. A pronounced lipophilic effect of EP was established toward olive oil and liquid paraffin. Cultivation of human keratinocyte cells (HaCaT) with crude EP and purified γ‐polyglutamic acid (PGA) showed higher viability than control group.

**Abbreviations**
DO: dissolved oxygen
EP: exopolymer
EPS: exopolysaccharide
*K_L_a*: volumetric mass transfer coefficient
OUR: oxygen uptake rate
PGA: γ‐polyglutamic acid
rpm: revolutions per minute
vvm: gas volume flow per unit volume medium per minute
*µ*_max_: maximum specific growth rate

## 1 INTRODUCTION

Microbial exopolysaccharide (EPS) producers can be found in different environmental niches including extreme ones. Despite the vast number of EPSs synthesized by extremophiles and the variety and complexity of their structures, they are still not competitive enough with plant polysaccharides mainly due to their high production costs, lower yields, and pricy downstream processes [1]. On the other hand, the variety in their structure and associated functional properties may provide high value applications in market niches that traditional polysaccharides obtained from plants and algae may not satisfy.

One of the main approaches to overcome the high market price is an establishment of the set of culture conditions that provide highest polymer yields. Physical factors, like agitation and aeration, are especially important parameters for aerobic fermentation processes in order to achieve maximal product accumulation in bioreactors. Agitation improves mass and oxygen transfer between the different phases, providing homogeneity of the culture liquid by continuous mixing. It also disperses the air into small bubbles to increase contact surface between two phases [2]. Intensive agitation could change and even damage the cells, while low‐speed agitation does not provide liquid homogeneity [3]. Another obstacle in bioreactor fermentations causing oxygen and mass transfer limitations could be a sharp viscosity increase of the culture liquid by the accumulated EPS [4].

Aeration provides continuous availability of oxygen in the culture liquid, contributes to its mixing and removes the gases produced during the process. Continuous oxygen supply is required in bioreactor for cultivation of aerobic microorganisms. Due to the low solubility of oxygen in liquid culture, it can be determined as a growth rate‐limiting factor for some microorganisms. Higher aeration rate provides better oxygen availability and appears to be favorable for an enhanced EPS production however it could also have a negative effect in some cases due to oxygen toxicity [5]. The oxygen availability for cells depends on the oxygen transfer rate (OTR) from the gas to the liquid phase, the rate of oxygen transport in the cells, and the oxygen uptake rate (OUR) by the microorganism. The efficiency of oxygen supply is characterized by the volumetric mass transfer coefficient, *K_L_a*, which estimation permits an effective scale‐up of bioreactors [6].

One of the most prospective properties of biopolymers is their use as emulsifiers, stabilizers, and foaming agents for cosmetic and food industries. Emulsifiers are adsorbed at the interface between the dispersed and continuous phase to stabilize the dispersed particles by means of their functional groups, spatial interactions, hydrophobic and hydrogen bonds. Bioemulsifiers are environmentally friendly and display higher selectivity, stability, and efficiency than many synthetic ones [7]. Industrial use of exopolymers (EPs) from halophiles suggests several advantages: these microorganisms are not pathogenic; their products exhibit enhanced stability toward other physicochemical conditions like high temperature, pH, and salinity, and good emulsifying and foaming ability [8]. Although several halophilic producers are known, still halophilic EPSs with an industrial significance are not known suggesting the fact that the diversity and biotechnological potential of salt niche habitats are largely unexplored. Some halophilic microorganisms synthesize EPs with protein fractions, namely glycoproteins, which enhance their emulsifying properties [9].

**PRACTICAL APPLICATION**

The manuscript suggests an effective bioreactor process for production of an exopolymer (EP) by a halophilic bacterium *Chromohalobacter canadensis* 28. The described properties determine its attractiveness for cosmetic industry. Valuable practical application elements referred to:

- Unusually high increase in EP yield after mass and air transfer optimization in a laboratory bioreactor referred the strain among the five best marine producers of EP.
- Fermentation bioreactor process for EP synthesis by *C. canadensis* 28 is very short, lasting only 48 h.
- High NaCl concentration in the medium could decrease contamination problems in industrial processes.
- Estimation of kinetic parameters of the process permits its scale‐up.
- EP expressed better emulsifying properties than commercial hydrocolloids.
- EP showed a good synergism with commercial hydrocolloids and improved its emulsifying properties.
- EP does not suppress viability of cell lines and could be successfully applied in cosmetic products.

In our previous studies, a halophilic bacterium *Chromachalobacter canadensis* was established to produce an unusual EP containing both EPS and γ‐polyglutamic acid (PGA) fractions and reported as the first halophilic bacterium able to produce PGA [10]. The limited knowledge on the effect of agitation and aeration on biomass and EP production by halophiles has determined the main aim of the current work, namely to study the EP biosynthesis by *C. canadensis* 28 in different mass and oxygen transfer conditions in a laboratory bioreactor. Estimation of the bioreaction kinetic parameters generates initial database for scale‐up of the process. In addition, the specific EP functional properties were analyzed in the light of feasibility for future application in cosmetic industry with the in‐vitro viability analysis of human keratinocyte cells.

## 2 MATERIALS AND METHODS

### 2.1 Microorganism

A halophilic bacterium *C. canadensis* 28 was isolated from a crystallizer pond of Pomorie salterns (42°35′33“N, 27°37′21″E), Burgas Bay, Black Sea, Bulgaria and deposited in NBIMCC (WDCM № 135) under the number 8924 [10]. The strain synthesized EP consisting of PGA (72% w/w) and EPS (14.3% w/w) fractions where the monomeric molar composition of the EPS was found to be: 36.7% glucosamine; 32.3% glucose; 25.4% rhamnose; 1.7% xylose, and 3.9% unidentified sugar.

### 2.2 Experimental setup and operation mode

The composition of the medium providing highest EP yield was determined in our previous study [10]. Colonies were transferred to 20 mL of culture medium in 100 mL shake flask and incubated for 72 h at 30°C, then the cells were transferred into 100 mL fresh medium and incubated for 20 h up to OD of 2‐2.5 at 660 nm. Bioreactor was inoculated with 3.0% v/v of an 18‐h culture under aseptic conditions. pH value was adjusted to 7.5 before sterilization and left to follow its natural course without control.

All experiments were carried out in a univessel 3‐L single impeller jacketed glass reactor (BioStat A, Sartorius, Germany) with an initial working volume of 1.5 L and internal diameter of 0.13 m. A moderate‐shear single hydrofoil six‐blade impeller Narcissus of diameter 0.053 m was used for agitation. Using impeller Narcissus provided mild stirring that decreased the cell stress and disruption of large biopolymer molecules and cells [11]. The temperature was maintained at 30°C by an external heating jacket. Precise temperature control was achieved with a solid‐state thermistor regulator providing a control accuracy of ±0.2°C. Foaming was regulated by the addition of Antifoam 204 (Sigma–Aldrich). The dissolved oxygen (DO) level (%) was measured in all experiments by an oxygen sensor (Endress+Hauser, Germany). The reactor was sterilized by autoclaving at 120°C for 20 min. The bioreactor was operated in batch mode for 48 h with constant temperature. The effect of agitation on growth and EP production was studied at 600‐1000 rpm through an interval of 100 rpm. The effect of aeration rate was studied at 0.5, 1.0, and 1.5 vvm, 900 rpm agitation. Culture broth samples (20 mL) were taken at 3‐h time intervals during the cultivation runs and used for measurement of the cell growth, EP quantification, estimation of EPS and protein, and substrate consumptions. Bacterial growth was monitored by measuring OD 660 nm. One unit OD was established to refer to 0.459 mg/mL dry biomass weight at cultivation parameters of 900 rpm and 1.0 vvm.

### 2.3 Measurement of oxygen uptake rate (OUR) and mass transfer coefficient (*K_L_a*)

The OUR and *K_L_a* were determined by the unsteady stop‐gassing measurement technique [12]. An oxygen probe was used, and the dynamic measurement was conducted in line with the probe dynamics. The OUR was determined from the depletion of the DO concentration ((*dC_0_*/*dt*)*_r_*) following the interruption of the air flow and the occurrence of net respiration (*r*). OUR was calculated from the equation:

(1) $${\left(dC_{0}/dt\right)}_{r}=\mathrm{OUR}.$$

At air flow restart with known OUR, *K_L_a* was determined from the overall oxygen balance of the absorption stage (*a*) following the integration of the equation:

(2) $${\left(dC_{O}/dt\right)}_{a}=K_{L}a\left(C_{O}^{*}-C_{O}\right)-\mathrm{OUR},$$

where ${\left(dC_{0}/dt\right)}_{a}$ is the accumulation of oxygen in the liquid phase and is $C_{O}^{*}$ is its equilibrium value.

The effects of agitation and aeration on the kinetic parameters on bench scale were studied. The values for OUR and *K_L_a* were determined in the exponential phase with two different conditions (1) at 0.5 vvm and 600‐1000 rpm through an interval of 100 rpm and (2) at agitation speed of 900 rpm at three aeration rates, namely, 0.5, 1, and 1.5 vvm.

All analyses were performed in triplicate, and the average values were presented. The standard error calculated from EP and biomass data deviation were 7% and 6%, respectively. The curves reflecting the changes of DO concentration, as a result of the experimental stopping of the gas fluid were integrated by parallel approximations at various time intervals along the two sections of the curves, that is, the respiration and the absorption one. As a result, the deviations' standard error was found to be 11% for OUR and 5% for *K_L_a*.

### 2.4 Extraction of the polymer fraction and analytical methods

Cells were removed in the late stationary phase of cultivation (48 h) and dry cell weight (DCW) was determined after culture centrifugation at 4000 × *g* for 20 min, the pellet was washed and dried to constant weight at 50°C. The cell‐free supernatant was preserved at −20°C for a determination of lactose concentration and for the quantification of the EP produced. EP fraction was recovered after precipitation with an equal volume of cold absolute ethanol. The pellet was washed twice in ethanol and dissolved in hot water. NaCl was removed from the supernatant by overnight dialysis against distilled water and then samples were dried at 50°C. The carbohydrate content was determined using the phenol/sulfuric acid method with glucose as standard [13]. Protein concentration consisting mainly from polyglutamic acid [10] was determined by Lowry test using bovine serum albumin as a standard. EP content was estimated as a sum from EPS and protein content. As the established values for nucleic acids content did not exceed 1%, they were not reported in the current work.

### 2.5 Synergistic effect of EP with commercial hydrocolloids

The synergistic effect was investigated by adding of 0.5% xanthan gum, guar gum, or cellulose gum (FLUKA, Switzerland) and EP in concentrations from 0.5% to 2%. The emulsifying properties were investigated with commercial sunflower oil as oil phase in ratio 1:1 according to the method described by Kuncheva et al. [14]. Emulsion activity of EP was also tested with other plants, minerals, and organic oils like olive oil, cocoa oil, silicone oil (cyclopentasiloxane DC 345), and liquid paraffin. The quality of the emulsion obtained was monitored by the dispersion indicator (determined spectrophotometrically by the light transmittance *T*, %) and the emulsion stability expressed as separated oil and water phase accounted after centrifugation. Model oil‐in‐water emulsions, that is, the water being the continuous phase, were prepared. After resolving of EP, the oil was added (1:1) and homogenization and emulsification were performed by using а disperser IKA ULTRA‐TURAX T 1 (IKA, Germany) at 3600 rpm. The quality of the obtained emulsions was determined by measuring the light penetration that reflects the heterogeneity degree of molecules in the mixtures. It was monitored by the percent translucency index (*T*, %) by using a spectrophotometer Camspec M 107 (Camspec Analytical Instruments, Camspec, UK) at wavelength of 540 nm. The emulsion stability was evaluated by measurement of the amount of residual oil and water phase separated after centrifugation of the emulsion at 3000 × *g* for 20 min. The dispersion characteristics of the emulsions (size distribution and average size of the droplets) were examined by means of Dynamic Light Scattering (DLS) that measures Brownian motion and relates it to the size of the particles [15]. The particles were illuminated by laser and intensity fluctuations in the scattered light were registered by Zetasizer Nano particle characterization system of Malvern [16]. The test samples were appropriately diluted (200 × when EP concentration was 0.5% and 300 × when EP concentration was 2%).

### 2.6 In‐vitro biocompatibility test

In‐vitro cell proliferation and viability assay, WST‐1 (4‐[3‐(4‐iodophenyl)‐2‐(4‐nitrophenyl)‐2H‐5‐tetrazolio]‐1, 3‐benzenedisulfonate) (Roche Applied Science, Germany), was performed for crude EP polymer and purified PGA polymer with human keratinocyte cell line (HaCaT). Polymers are sterilized by 0.22 µm syringe filter and HaCaT cells at the 70% confluence were seeded onto 96‐well cell culture plates at the density of 5 × 10^3^ and incubated for 24 h. After cellular attachment, complete media (10% FBS and 1% penicillin‐streptomycin [PAN Biotech, Germany]) was replaced with 0, 10,100, 500, and 1000 µg/mL crude EP and PGA containing media and cells were treated for 24, 48, and 72 h at 37°C in a humidified incubator that contained 5% CO_2_. WST‐1 cell proliferation reagent was added onto wells at the end of incubation periods and incubated for two more hours at 5% CO_2_ containing dark incubator at 37°C, and absorbance was measured at 450 nm by Multimode plate reader (GloMax Multi + Microplate Multimode Reader, Promega, USA). Control group did not contain any polymer and considered as 100% viable. Statistical analysis of data was performed by Graph Pad Prism 5 using One‐Way Anova and Tukey tests. All experiments were performed in triplicate and a *p* value above 0.05 was considered as statistically significant.

## 3 RESULTS AND DISCUSSION

### 3.1 The effect of agitation on microbial growth and EP production

**FIGURE 1:**
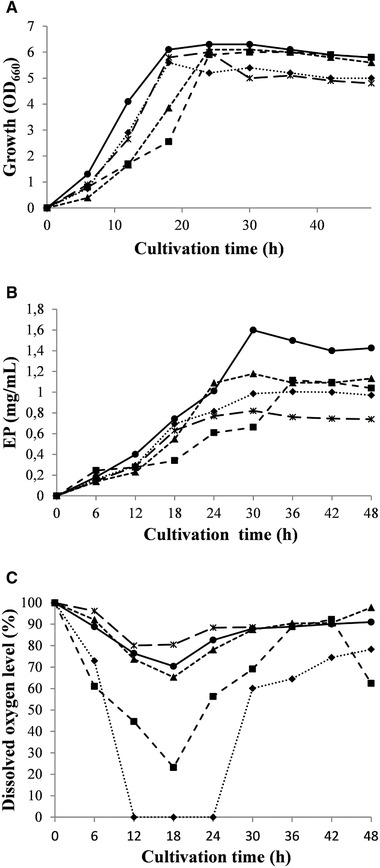
Effect of different agitation rates on *C. canadensis* 28 growth (A), EP (B), and dissolved oxygen level (C) at aeration of 0.5 vvm. (♦), 600 rpm; (■) 700 rpm; (▲), 800 rpm; (●), 900 rpm; (ж), 1000 rpm

Several factors influence mixing and mass transfer in stirred bioreactors like the scale and type of bioreactor, the type of stirrers, the stirrer speed, the gas flow rate used, the presence of biomass and polymers synthesized by it [12]. Bioreactor cultivation for investigation of the agitation influence on cell growth and EP production was performed at а constant aeration rate of 0.5 vvm and agitation speeds of 600, 700, 800, 900, and 1000 rpm. A lag phase lasted of about 6 h for all rates, followed by an exponential phase until 18‐24 h of cultivation time and a stationary phase until 48 h, as monitored by cease of cell growth. Such a way, the polymer production was shorten twofold in comparison with batch cultures (96 h) [10]. Growth of *C. canadensis* 28 varied in OD_660_ range 5.6 (600 rpm) and 6.2 (900 rpm) (Figure 1A). The reached maximum specific growth rates (*µ*_max_) were also similar for different speeds and varied in the interval 0.18‐0.24 h^−1^. The carbon source lactose decreased with an almost equally fast consumption rate during exponential and early stationary phase reaching values in the range 2.9‐3.4 mg/mL. The EP content, determined as a sum of EPS and protein contents was 1.5‐fold higher at 900 rpm than at 600 rpm (1.497 and 1.004 mg/mL, respectively) (Figure 1B). A steady increase in EP level observed when agitation speed increased from 600 to 900 rpm, seemed to be caused by an improved mass transfer and the established sharp decrease at 1000 rpm could be assigned to the mechanical stress of cells at highest agitation. Very different DO concentration profiles were observed for the various agitation speeds (Figure 1C) and minimal oxygen levels in the liquid varied from 0% (600 rpm) to 80.1% (1000 rpm). Oxygen limitation was observed at 600 rpm although DO provided sufficient growth. Further increase of the agitation speed resulted in oxygen excess but did not influence the microbial growth significantly. Under conditions providing highest EP yield (900 rpm), the reached minimal DO level was 70.4%.

### 3.2 Effect of aeration on microbial growth and EP production

**FIGURE 2:**
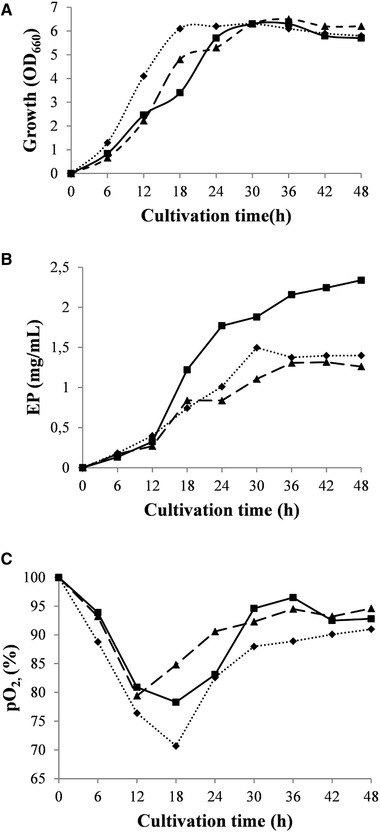
Effect of different aeration rates on *C. canadensis* 28 growth (A), EP (B), and dissolved oxygen level (C) at agitation of 900 rpm. (♦), 0.5:1 vvm; (■) 1:1 vvm; (▲), 1.5:1 vvm

The effect of aeration rate on EP production was investigated at three aeration rates, 0.5 vvm (0.75 L/min), 1 vvm (1.5 L/min), and 1.5 vvm (2.25 L/min) at the agitation speed that ensured maximum EP production (900 rpm) (Figure 2). Similar to the agitation influence on cell growth, the difference in OD_660_ values was not significant and varied between of 6.1 (0.5 vvm) to 6.5 (1.5 vvm) (Figure 2A). The culture passed through 6 h lag phases and exponential phases lasting for 18 h (0.5 vvm) and 30 h (1.5 vvm). The slower growth of the strain at higher aeration rate could be explained by a mechanical stress of air flow on the cells. The EP yield of 1.50 mg/mL at 0.5 vvm increased up to 2.34 mg/mL at 1.0 vvm and decreased about 1.8‐fold to 1.3 mg/mL at 1.5 vvm (Figure 2B). The DO was in excess in all processes and varied between 70.7% and 84.8% (Figure 2C). Comparing the performance of the particular system with reference data, the bioreactor behavior in the specific case appeared to be similar to the one described by Garcia–Ochoa et al. [6]. Referring to the specific biosynthesis, these authors observed that the increase of DO concentration favored the polymer xanthan production rate. The positive effect of an improved oxygen supplementation was also reported for EPS‐R from marine bacteria *Halomonas xianhensis* and *Hahella chejuensis* SUR308 [17, 18]. Similar to the results obtained for a glycoprotein by *Streptomyces kanasenisi* [3], the effect of aeration rate on EP production by *C. canadensis* 28 was more significant than agitation speed. On the other hand, higher effect of agitation on EPS synthesis was reported for EPS from a thermophilic bacterium [19].

**FIGURE 3:**
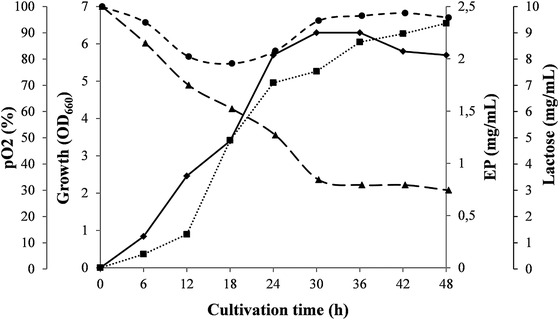
Time course of *C. canadensis* 28 growth (♦), EP content (■), dissolved oxygen level (●), and lactose depletion (▲) at agitation of 900 rpm and aeration of 1:1 vvm

Time course of microbial growth, EP production, DO concentration, and substrate depletion at optimal agitation and aeration conditions are presented in Figure 3. The kinetics of EP production by *C. canadensis* 28 was similar to that of EPS produced by *Salipiger mucosus* A3T [20] and revealed intensive synthesis during the exponential phase but persisted during the stationary phase with a lower yield. The registered decrease of substrate lactose with an almost equal fast consumption rate was observed during exponential and early stationary phase reaching value of 3.0 mg/mL in the cultural broth. Residual almost constant quantity of lactose was observed in all processes suggesting that carbon source is not a growth limiting factor. Its consumption in the early stationary phase without display of substantial cell growth probably served for cells’ maintenance. The registered decrease of lactose in the early stationary phase without display of substantial cell growth was observed for other Gram‐negative bacteria [21]. In aim to enhance EP yield, Tween 80, in a concentration of 0.1 mg/mL, was added on 24‐h cultivation and 32% EP enhances up to 3.085 mg/mL was registered without significant changes of other measured parameters. Some authors have explained the additional improvement of EP synthesis in presence of surfactants by affecting the rheological properties of the liquid and enhancing the oxygen concentration [22]. Investigations performed in the current study revealed a high effect of the agitation and aeration on EP production and twofold higher production (up to 2.34 mg/mL) in comparison with the EP yield of 1.2 mg/mL in shaking flasks [10] was observed reaching a degree of increase not reported before by other authors. This ratio is significantly higher than EPS production by *H. xianhensis* SUR308 for which an increase after optimized aeration was from 2.55 mg/mL to 2.85 mg/mL [23]. The reached EP yield of 3.085 mg/mL was three times more than the observed average production yield for marine EPS of around 1 mg/mL [24]. The results for EP synthesis by marine bacteria in laboratory fermenters summarized in Table 1 revealed that *C. canadensis* 28 was among the six strains with production levels exceeding 1 mg/mL. Comparison of the results in the table revealed significant advantages of EP production by *C. canadensis* 28. Growth at highest reported NaCl concentration (15%) prevented microbial contamination; short fermentation process lasted only 48 h; good ratio of product yield (*P*) and utilized carbon source (*S*) was established. Highest productivity in a bioreactor by a marine microorganism has been reported for *Halomonas smyrnensis* producing 18.06 mg/mL EPS in 100 h lasting process [25]. The process with *Pseudoalteromonas sp*. was shorter (17 h) than that with *C. canadensis* 28, however, it has performed at 0% NaCl [27].

**TABLE 1:** Highest levels (more than 1%) of exopolymer synthesis in a bioreactor by marine microorganisms

Producer	Process duration (h)	Carbon source used (%); salt (%)	Yield (mg/mL)	Yield (*P/S*, %)	References
*Halomonas smyrnensis*	100	Sucrose, 5; NaCl, 13.7	18.06	36.1	[25]
*Hahella chejuensis*	120	Sucrose, 2; NaCl, 1	9.23	46.2	[26]
*Pseudoalteromonas sp*.	17	Glucose, 3; NaCl, 0	4.4	14.7	[27]
*Saccharophagus* *degradans*	72	Glucose, 1.5; NaCl, 2.3	4.12	27.5	[28]
*Chromohalobacter canadensis* 28	48	Lactose, 1.0; NaCl, 15	3.08	30.8	This study
*Halomonas* sp. AAD6	96	Sucrose, 5; NaCl, 13.7	1.84	3.7	[29]

### 3.3 Effects of agitation and aeration rates on OUR and *K_L_a* on bench scale

**FIGURE 4:**
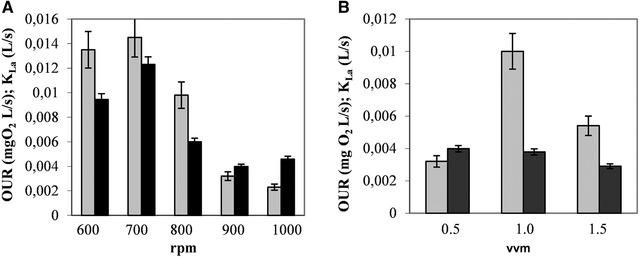
Oxygen transfer parameters OUR and $K_{L}a$ versus: (A) mixing intensity, rpm; (B) aeration rate, vvm. Parameters were estimated during exponential phase. Symbols: 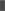, OUR; 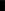, $K_{L}a$

As the oxygen transfer parameters, the OUR and $K_{L}a$ are the most appropriate estimates for bioprocess extrapolation and scale‐up, which were determined and analyzed in our work (Figure 4A,B). Like a heteropolysaccharide by *Salipiger mucosus* A3T [20], the polymer production by *C. canadensis* 28 was characterized by a dynamic kinetics during the exponential phase and persisted during the stationary phase with negligible changes of the parameters. Considering agitation rates between 600 and 1000 rpm at 0.5 vvm, OUR values in the exponential phase vary in the range 14.5 × 10^−3^ (700 rpm) to 2.3 × 10^−3^ (1000 rpm) mg O_2_/L per second and *K_L_a* values pass through a maximum of 12.3 × 10^−3^/s at 700 rpm and a minimum of 3.98 × 10^−3^/s at 900 rpm (Figure 4A). On the other hand, the *K_L_a*‐values were seen to be comparable at 0.5 and 1.5 vvm (Figure 4B).

Comparison of these results with the results from Figures 1 and 2 revealed that the EP production rate corresponded to *K_L_a* evolution. The mass transfer performance parameters for the highest productivity fermentation obtained at 900 rpm and 1 vvm showed low values of *K_L_a* of 3.98 × 10^−3^/s and OUR of 10 × 10^−3^ mg O_2_/L.s. Further increase of impeller speed beyond 900 rpm, for example, up to 1000 rpm at 1 vvm led to retention of *K_L_a* and OUR fall. As a result, biomass decrease and fall in EP production were observed. While not knowing the exact reason of the decline, one could presume that the gradual increase of hydrodynamic stress in the biofluid causes metabolic stress on EP production and/or EP accumulation in the medium could hinder the air bubble dispersion in the jar [17]. A negative effect on the final EPS production at high *K_L_a* has been observed for a glycoprotein by *S. kanasenisi* strain ZX01 [3].

### 3.4 Emulsifying activity and synergistic action of EP from *Chromohalobacter canadensis* 28

Investigations of the emulsifying properties of trade hydrocolloids and EP from *C. canadensis* 28 when applied alone revealed superiority of EP emulsion (Table 2). Emulsion stability for used trade hydrocolloids varied from 4% to 50% while it was 70% for EP. The oil‐phase separation was also significant (up to 58% for xanthan gum). Oil phase was not separated for both, EP from *C. canadensis* 28 and guar gum. The emulsifying properties of trade hydrocolloids were significantly improved when halophilic EP was added at different EP concentrations and reached 78‐100% emulsion stability. The emulsions containing 1.0% EP and 0.5% cellulose or xanthan formed 100% emulsions and neither water nor oil separated phases were observed after centrifugation. As previously reported, a similar emulsifying effect of 100% emulsion stability was achieved at EP concentration of 1.5% alone [10]. However, the emulsion containing 0.5% EP and 0.5% cellulose had higher stability (93.6%) than emulsion containing 1% EP alone (78%) or 0.5% xanthan and 0.5% EP (78%) demonstrating the good synergism with cellulose. At almost all concentrations tested for mixed emulsion systems, the percentage of oil separation was zero and it was insignificant only for 0.5% guar gum. Water released in the stability test was also greatly reduced in a presence of 0.5% EP and varied in the range 6‐22%. Release of 6% water was observed at 0.5% guar gum + 1% EP (a total polymer presence of 1.5%), while at 1.5% EP alone, emulsion stability was 100% suggesting that guar gum even deteriorates emulsion properties of EP. In terms of dispersion, the lowest *T*‐index values were observed in the experiments with cellulose gum and EP that confirmed good synergic action among two polymers. These values were lower than the *T*‐values obtained when using EP alone. The *T*‐values for other hydrocolloids were higher, such a way reflecting reduced stability of the emulsions. These results clearly demonstrated good emulsifying properties of EP alone and in combination with cellulose and its good prospect for exploration as an emulsion supplement in industry. Probably, the eminent role of EP from *C. canadensis* 28 in the oil emulsification is connected with the presence of considerable amount of PGA in the molecule (72% w/w). Emulsifying efficiency at low concentrations of bacterial polymers composed of glycoproteins, lipopolysaccharides, and lipoproteins have been reported by Bramhachari et al. [30]. Good emulsification properties have been reported for EPs synthesized by *Streptomyces* sp. ZX01 [31] and by *Halomonas* sp. strain TG39 [9]. About 60% of the EP synthesized by *Streptomyces* sp. ZX01 represented a protein fraction while that from *Halomonas* sp. strain TG39 reached up to 31.8%.

**TABLE 2:** Emulsion stability of the oil/water emulsions achieved with 0.5% hydrocolloid and different EP concentrations added in the water phase

		Separated phase (%)			
Hydrocolloids	EP concentration (%)	Oil	Water	Emulsion	*Т* (%)
EP from *C. canadensis* 28	0.5	0	30	70	44.0
Guar gum	0	0	50	50	66.8
	0.5	2	14	84	47.6
	1.0	0	6	94	34.6
	1.5	0	3	97	34.4
	2.0	0	0	100	25.6
Cellulose gum	0	23	33	44	22.6
	0.5	0	6.4	94	28.3
	1.0	0	0	100	18.5
Xanthan gum	0	58	38	4	54.6
	0.5	0	22	78	64.6
	1.0	0	0	100	34.2

Concentration (in %) refers to the content in the water phase mixed with the oil phase (1:1).

Different oils mainly from plant and synthetic origin were used as traditional components in many cosmetic products. One plant, one mineral, and one organic oil were chosen for further characterization of the emulsion activity of EP from *C. canadensis* 28 in oil/water emulsions when sunflower oil was replaced by other oils (Table 3). The pronounced lipophilic effect of 0.5% EP was demonstrated by a lack of recovered spinning oil for all experiments except cocoa butter where a negligible amount of oil was separated. Significantly higher quantities of an EP from *Halomonas* sp. S19 (5‐10%) stabilized the emulsions with coconut oil and paraffin [32]. The most stable emulsions (90‐100%) were formed with sunflower oil, olive oil, and silicone oil and separated water phase was not observed for sunflower and silicone oil when 2% EP concentration was used. Good emulsifying activity of EPS synthesized by *S. mucosus* strain A3T has been established also with sunflower oil and olive oil although crude oil has been the best compound for oil phase [20]. The emulsifying properties of EPS 71a produced by *Enterobacter cloacae* has been comparable to commercial gums [33]. Good emulsion stability after adding of EP from *C. canadensis* 28 correlated with low *T*‐index values measured for these model emulsions. Weaker emulsion in cocoa butter emulsions could be explained by the solid consistency of cocoa butter at room temperature and mixed zones of emulsion/oil and emulsion/water were observed after centrifugation due to not enough homogenous consistency of this emulsion. However, the lowest *T*‐index of 16.5% was measured in this final emulsion suggesting that a good quality emulsion could be used in cosmetic products even with cocoa oil after removing of separated phases.

**TABLE 3:** Emulsifying activities of EP from *C. canadensis* 28 for different oils

		Separated phase after centrifugation, %				
Oil phase, 50%	EP concentration (% in water phase)	Oil	Water	Emulsion	*Т*, %	Average emulsion size of particles, d (nm)
Sunflower oil	0.5	0	30	70	44.0	5295
	2.0	0	0	100	22.6	n.d.
Olive oil	0.5	2	40	58	34.3	5968
	2.0	0	10	90	21.4	7464
Cocoa oil	0.5	10	40	50	53.5	n.d.
	2.0	drops	40	60	16.5	n.d.
Silicone oil (Cyclopentasiloxane DC 345)	0.5	drops	10	90	55.0	6983
	2.0	0	0	100	26.8	19 471
Liquid paraffin	0.5	drops	40	60	52.8	5404
	2.0	0	24	76	36.3	5949

Maintaining an emulsion requires a presence of emulsifying agent able to decrease the surface tension of the droplets and to form a barrier for preventing coalescence of droplets. Best dispersion characteristics measured by dynamic light scattering (DLS) were obtained with olive oil and liquid paraffin containing 0.5% EP. These emulsion systems had smaller oil droplets and were homogeneous in the distribution of oil particles (contained only one peak of size distribution by intensity) (data not shown).

### 3.5 In‐vitro biocompatibility results

**FIGURE 5:**
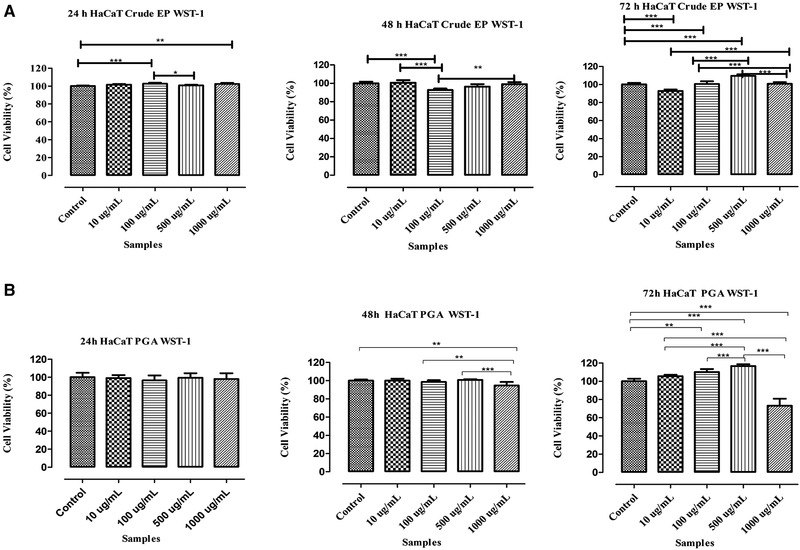
Viability results of HaCaT (Human Keratinocyte Cell line) cells after being cultivated with crude EP (A) and purified PGA (B). (A p value below 0.05 is represented as: *; 0.01 to 0.05; **, 0.001 to 0.01; and ***, <0.001)

Viability results of HaCaT cells after being cultivated with crude EP and purified PGA for 24, 48, and 72 h are shown in Figure 5A,B. According to WST‐1 cell proliferation analysis, cellular viability at the end of 24 h were 100%, 101.6%, 102.8%, 100.7%, and 102.4% for control, 10, 100, 500, and 1000 µg/mL samples, respectively. Viability after 48 h cultivation changed to (%): 100, 100.6, 92.78, 96.44, 99.04, and after 72 h it was 100, 92.81, 100.6, 109.5, and 100.8. Crude EP group showed higher viability than control group for 24 h and viability did not decrease below 80% for 48 and 72 h even at the highest doses it exceeded the control after 72 h. For the purified PGA viability results for the first 24 h was recorded as: 100%, 99.15%, 96.46%, 99.36%, 98.44% while 100%, 100.1%, 98.47%, 100.8%, 94.57% viability was observed at the end of 48 h. Although all doses of samples for 24 and 48 h showed almost similar viability with control group, at the end of 72 h viability of cells increased to 105.6%, 110.2%, and 116.2% for the doses of 10, 100, and 500 µg/mL while control group showed 100.00% viability. Su et al. [34] has investigated toxicity, genotoxicity, and viability effect of ɣ‐PGA containing mouthwash with mouse embryonic fibroblasts (3T3) and Chinese hamster ovary cells. No genotoxicity and cytotoxicity has been observed while 98% cell viability has been measured with antibacterial activity and this product was proposed as an alcohol‐free mouthwash without any cytotoxicity. γ‐PGA‐ and ε‐polylysine (ε‐PL)‐based pH sensitive poly (amino acid) hydrogels have been prepared by Hua et al. [35] and high biocompatibility (117.6%) has been observed with in‐vitro cell culture test with murine fibroblast cells (L929). According to keratinocyte cell viability results, purified γ‐PGA showed increased viability in dose–dependent manner for 10, 100, and 500 µg/mL at the day 3 which is higher than control group. Similar result is also observed for crude EP at a dose of 500 µg/mL for the day 3. In conclusion, PGA and EPS combined in the crude EP polymer can be a promising candidate for cosmeceutical applications with increased keratinocyte cell viability and proliferation.

## 4 CONCLUDING REMARKS

This study is among the low number investigations on the kinetics EP production by halophiles and the first report of the ability of a species from the genus *Chromohalobacter* to grow and produce polymer in a stirred bioreactor in a highly efficient process. As a result of mass and aeration optimization, an unusual increase of EP yield in a fermenter was observed (more than twofold) and such a way *C. canadensis* 28 referred among the best halophilic producers of EP. Bioreactor processes with exploration of this microorganism has a combination of advantages that determines its superiority, like a short fermentation process in a high NaCl concentration. Its valuable emulsifying and biocompatible properties point to the prominent potential of this polymer for cosmetic and biomedical exploration.

## CONFLICT OF INTEREST

The authors declared no conflict of interest.
